# The High-Throughput Screening of Microorganisms to Eliminate Ethyl Carbamate in Chinese Liquor

**DOI:** 10.3390/foods13060864

**Published:** 2024-03-13

**Authors:** Zirui Yin, Jianghua Li, Jian Chen, Guocheng Du, Xinrui Zhao

**Affiliations:** 1 Science Center for Future Foods, Jiangnan University, 1800 Lihu Road, Wuxi 214122, China; 6210201054@stu.jiangnan.edu.cn (Z.Y.); lijianghua@jiangnan.edu.cn (J.L.); jchen@jiangnan.edu.cn (J.C.); gcdu@jiangnan.edu.cn (G.D.); 2 Key Laboratory of Industrial Biotechnology, Ministry of Education, School of Biotechnology, Jiangnan University, 1800 Lihu Road, Wuxi 214122, China; 3 Jiangsu Province Engineering Research Center of Food Synthetic Biotechnology, Jiangnan University, 1800 Lihu Road, Wuxi 214122, China; 4 Engineering Research Center of Ministry of Education on Food Synthetic Biotechnology, Jiangnan University, 1800 Lihu Road, Wuxi 214122, China; 5 Key Laboratory of Carbohydrate Chemistry and Biotechnology, Ministry of Education, Jiangnan University, 1800 Lihu Road, Wuxi 214122, China

**Keywords:** ethyl carbamate, urea, cyanide, Chinese liquor, high-throughput screening, heterogeneous expression, simulated fermentation

## Abstract

Ethyl carbamate (EC) is a 2A classified carcinogen in Chinese liquor that has raised many problems regarding food safety. Applying microorganisms to control the content of EC precursors in fermented grains has been proven as an effective method to reduce EC in alcoholic beverages. However, the utilization of microorganisms to decrease the precursors of EC (urea and cyanide) is still incomplete in regard to Chinese liquor. Thus, it is necessary to isolate strains with the degradative activities of urea and cyanide. Herein, *Bacillus sonorensis* F3 and *Bacillus licheniformis* YA2 strains were isolated from the fermented grains through multiple rounds of high-throughput screening, and the degradative abilities in urea and cyanide reached 95.72% and 75.48%, respectively. In addition, the urease from the *B. sonorensis* F3 strain and the carbon nitrogen hydrolase from the *B. licheniformis* YA2 strain were identified by the heterogeneous expression in *Escherichia coli*. Then, both F3 and YA2 strains were combined at a ratio of 5:1 and applied to eliminate the EC in the simulated fermentation of Chinese liquor; as a result, 51.10% of EC was reduced without affecting the main composition of flavor substances. The obtained strains have great potential in terms of the improvement of quality and safety of Chinese liquor.

## 1. Introduction

Chinese liquor features in a range of representative distilled spirits which are popular worldwide for their unique flavor. However, a classified 2A carcinogen, ethyl carbamate (EC), exists in Chinese liquor and has caused many problems in terms of food safety [1,2]. After being absorbed by the human body, EC is converted to epoxides that lead to DNA adducts and further induce the formation of lung tumors [2,3]. It has been observed that an increasing trend of carcinoma in mice was caused by the intensified distribution of EC with ethanol [4]. Based on the detection of EC in Chinese liquor, its concentration could reach 192 μg/L [5], which is higher than the limited standard of EC for distilled spirits in Canada, Brazil and Japan (150 μg/L) [6], thereby posing a great threat to public health. Thus, it is necessary to reduce the concentration of EC in Chinese liquor.

**Figure 1:**
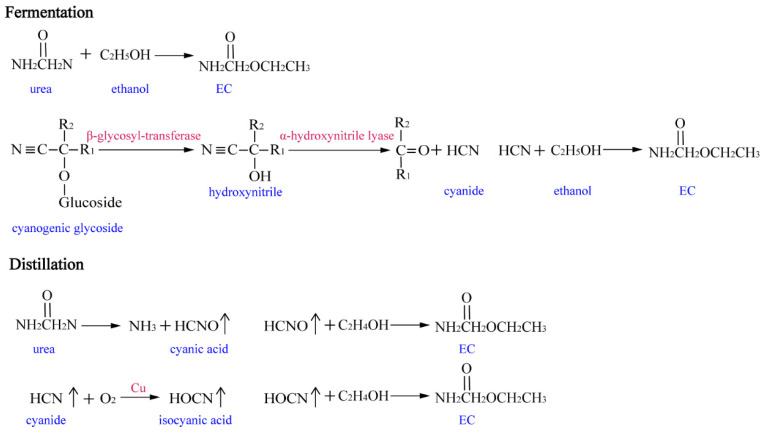
The forming process of EC through a urea and cyanide pathway. The blue and red words represent substances and catalysts for the reactions, respectively.

The manufacturing of Chinese liquor consists of solid-state fermentation (SSF) and distillation. The process of SSF involves a variety of microorganisms to form ethanol and other flavors, while the process of distillation can form the base liquor [7]. According to the previous research, EC is mainly formed by the reaction of ethanol with precursors (urea and cyanide) during the distillation of Chinese liquor [6]. For the source of cyanide, it can be generated from cyanogenic glycosides in the raw materials (sorghum, etc.) by the catalysis of β-glycosyl-transferase and α-hydroxynitrile lyase during fermentation and storage [1,8]. As for urea, it primarily comes from the fermented grains. During the steam state of distillation at high temperature, urea can be converted into the vapor of cyanic acid, and a portion of cyanide can be oxidized into the vapor of isocyanic acid [9]. Then, the vapors of cyanic acid and isocyanic acid react with ethanol and sequentially form EC in the distilled base liquor [10] (Figure 1). However, due to the unique process of making Chinese liquor and complicated metabolic flux of the involved microorganisms, the mechanism of EC formation in Chinese liquor is still not clear, which makes it a challenge to eliminate EC.

At present, three strategies have been applied to eliminate EC in alcoholic beverages. Among them, the optimization of the manufacturing process has been proven as an effective approach to controlling the EC content in Chinese liquor, including in the double distillation, low temperature fermentation and slow distillation processes [11,12]; however, these methods usually have adverse effects on the unique flavor of Chinese liquor. Another strategy is the direct degradation of EC. For example, a *Lysinibacillus sphaericus* MT33 strain isolated from fermented grains of sesame-flavor Chinese liquor can reduce 41.77% of EC in fermented grains at the end of fermentation [13]. As for the control of EC precursors (urea or cyanide), this strategy is more important for the elimination of EC because a large proportion of EC in the base liquor is mainly generated from its precursors during the distillation at high temperature [14]. For example, the knockout of the *CAR1* gene in *Saccharomyces cerevisiae* can reduce the formation of urea and EC in wine [15]. In addition, acid urease was added to degrade urea and reduced 69.9% of EC in the fermented grains at the end of fermentation [16]. Moreover, a *S. cerevisiae* MT-1 strain was isolated and applied to degrade 46.45% of cyanide during the simulative fermentation of Chinese liquor [17]. Thus, it is an ideal and efficient method for reducing EC through applying microorganisms with the degradative activity of EC precursors, though the current reported microorganisms that can degrade cyanide were mainly isolated from wastewater or soil [18,19,20,21]. There are few reports on the in situ isolation of strains from fermented grains in terms of the degradative activity of cyanide. Therefore, there is an urgent need for obtaining microorganisms to reduce cyanide and EC in Chinese liquor.

In this study, the high-throughput screening methods were established to isolate strains with the degradative activities of main precursors to EC (urea and cyanide) from fermented grains. Through multiple rounds of screening, the strains showing the highest degradation rate of urea and cyanide (*Bacillus sonorensis* F3 and *Bacillus licheniformis* YA2) were isolated from the fermented grains, and the degradative abilities in urea and cyanide reached 95.72% and 75.48%, respectively. In addition, the related functional enzymes that can degrade urea or cyanide were identified through the heterologous expression in *Escherichia coli*. Then, the degradation of EC precursors and the elimination of EC was verified in the simulated fermentation system of Chinese liquor. The screening methods, functional microorganisms and related enzymes obtained in this study paved a practical way towards the elimination of EC to enhance the quality and safety of Chinese liquor.

## 2. Materials and Methods

### 2.1. Materials and Media

Fermented grains were obtained from a Chinese liquor distillery. The dry yeast for brewing was purchased from Angel Yeast Co., Ltd. (Yichang, China). The standard substances for the analysis of cyanide in water were purchased from the Tanmo Quality Inspection Technology Co., Ltd. (Beijing, China). It is composed of KCN dissolved in 0.1 mol/L NaOH. All solvents and reagents were analytical grade and purchased from Sinopharm Chemical Reagent Co. (Shanghai, China). The chloramine-T and 9-hydroxyxanthene were produced by McLean Reagent Co. (Shanghai, China).

M9 medium: 20 g/L glucose, 6.8 g/L Na_2_HPO_4_·7H_2_O, 3 g/L KH_2_PO_4_, 0.5 g/L NaCl, 0.8 g/L MgSO_4_·7H_2_O [22,23]. LB medium: 10.0 g/L peptone, 5.0 g/L yeast extract, 10.0 g/L NaCl. Minimal medium: 20.0 g/L glucose, 1.0 g/L yeast extract, 6.8 g/L Na_2_HPO_4_·7H_2_O, 3.0 g/L KH_2_PO_4_, 0.5 g/L NaCl, 0.01 g/L CaCl_2_, 1 mL of trace metal solution (H_3_BO_3_ 30 mg/L; CoCl_2_·6H_2_O 200 mg/L; ZnSO_4_·7H_2_O 100 mg/L; MnCl_2_·4H_2_O 10 mg/L; pH 6.0) and increasing content of cyanide (25 mg/L, 100 mg/L, 200 mg/L), pH 8.0 [24]. Glucose inorganic salt medium (GI): 50.0 g/L glucose, 10.0 g/L peptone, 5.0 g/L (NH_4_)_2_SO_4_, 2.0 g/L KH_2_PO_4_, 1.0 g/L MnCl_2_, 5.0 g/L NaCl, pH 7.0–7.2. NB medium: 10.0 g/L peptone, 3.0 g/L beef extract, 5.0 g/L NaCl, pH 7.2 ± 0.2. MRS medium: 52.2 g/L MRS broth medium [25,26,27].

### 2.2. Isolation of Urea-Degradation Microorganisms

To start, 3 g of each sample was inoculated into 10 mL of M9 medium for the enrichment containing an increasing content of urea (1.5 g/L, 3.0 g/L, 5.0 g/L) in a 50 mL centrifuge tube. M9 medium was sterilized at 115 °C for 20 min, and then filter-sterilized urea was added. To enrich aerobic and anaerobic urea-degradation microorganisms, it was incubated on a rotary shaker at 220 rpm, 37 °C for 48 h, and, in the anaerobic workstation, (Don Whitley Scientific, Bingley, British) at 30 °C for 5 days, respectively. The enriched sample was diluted 5 times and inoculated with M9 agar medium containing 5 g/L urea and incubated at 37 °C and anaerobic workstation at 30 °C, respectively. Microorganisms with the activity of urea degradation were enriched and adaptively evolved through continuous incubation with the increasing concentration of urea.

### 2.3. Identification of Urea-Degradation Microorganisms

Primary screening: All individual colonies grown in the M9 agar medium were sub-cultured by Qpix420 (Molecular Devices, San Jose, CA, USA) into 96-deep-wells containing M9 medium with 5.0 g/L urea and 6.0 mg/L bromcresol purple. A blank medium without microorganisms was used as control. Degradation of urea is a process of producing ammonia which alkalinizes the system and turns the color into purple. The purple isolates were selected for the secondary screening. The diacetylmonoxime spectrophotometry method was also applied to detect the rest content of urea in the 96-wells [28]. The isolates showed the lower rest content of urea were selected into the secondary screening.

Secondary screening: The screened isolates, after primary screening, were inoculated in 10 mL of LB medium at 30 °C for overnight culture. After ultrasonic cell-break, the rough extraction was used to determine the activity of urease by Berthelot colorimetry [29]. The rough extraction was boiled for 10 min and used as control, respectively. Isolates with higher activities of urease were used for the degradation of urea. The activity of urease was defined as follows: one unit is the activity of degraded urea to produce 1 μM ammonium per minute at 37 °C, atmospheric pressure and pH 5.5.

### 2.4. Screening of Cyanide-Degradation Microorganisms

Enrichment of strains: 3 g of fermented grains were inoculated into 10 mL of minimal medium containing increasing content of cyanide (25 mg/L, 100 mg/L, 200 mg/L), pH 9.0. To enrich the aerobic and anaerobic cyanide-degrading microorganisms, it is incubated on a rotary shaker at 220 rpm, 37 °C for 48 h, and at the anaerobic workstation at 30 °C for 5 days, respectively. Then, the enriched samples were inoculated on minimal agar medium containing 25 mg/L cyanide, then were inoculated in an incubator at 37 °C and anaerobic workstation at 30 °C, respectively.

Primary screening: After the enrichment, the microorganisms that could utilize cyanide as a nitrogen source were isolated. All isolates on the minimal agar medium were selected by Qpix420 (Molecular Devices) into 96-deep-wells in minimal medium containing 100 mg/L cyanide as the sole nitrogen source. In the following, individuals were sub-cultured into fresh minimal medium containing lower cyanide (50 mg/L) as the sole nitrogen source. As the concentration of cyanide in fermented grains fluctuated between 2.0–8.0 mg/L [14], the decreasing concentration of cyanide enabled the isolates to adapt to the toxicity of cyanide and the insufficient nitrogen source; thus the isolates that could utilize the lower concentration of cyanide as a nitrogen source were further isolated. Isolates that can grow in 50 mg/L cyanide were sub-cultured into 96-deep-well plates containing LB medium. The isolates reacted with cyanide in the form of whole-cell catalysis, and the content of ammonia in the system was detected. The o-phthalaldehyde (OPA)-trichloroacetic acid (TCA) spectrophotometry was used to assess the degradative effect of cyanide for the microorganisms [30,31].

Secondary screening: Primary screened isolates were incubated in LB medium containing 2.0 mg/L cyanide. Then, the degradation of cyanide was determined at 72 h and the strains with a high degradative effect on cyanide were selected.

### 2.5. Classification of Selected Strains

The selected bacteria were identified using 16S rDNA gene sequencing analysis. The genome was extracted and used as the template, and the universal primers were designed to amplify the 16S rDNA gene (27F: 5′-AGA GTT TGA TCC TGG CTC AG-3′, 1492R: 5′-TAC GGT TAC CTT GTT ACG ACT T-3′) [32,33]. PCR reactions were performed in 50 μL volumes containing 25 μL of Fastpfu Fly PCR supermix (Transgen Biotech, Beijing, China), 1 μL of each primer, 1 μL of genomic DNA and 22 μL of sterilized H_2_O. The PCR program was as follows: 98 °C for 1 min, 35 cycles of 98 °C for 10 s, 55 °C for 5 s, 72 °C for 30 s and a final extension of 72 °C for 1 min. PCR products were purified by a PCR purification kit (Transgen Biotech, Beijing, China), and amplicons were then sequenced. Sequence similarities were identified using the BLAST program at the National Center for Biotechnology Information (NCBI) website. The evolutionary history was inferred using the neighbor-joining method [34]. The evolutionary distances were computed using the p-distance method [35]. Evolutionary analyses were conducted by MEGA11 [36].

### 2.6. Analytical Method for EC and Its Precursors

Urea-degradation isolate F3 was cultured in different conditions containing 10 mg/L urea on a rotary shaker or an incubator at 220 rpm, 37 °C for 48 h. Before testing, 10 g of solid fermented grains were dissolved in ultra-pure water and then centrifuged after ultrasonic treatment for 15 min. The supernatant was taken for testing. The concentration of urea was determined by high performance liquid chromatography–flame ionization detection (HPLC-FID) (Agilent, Santa Clara, CA, USA) with a C18 column (4.6 mm × 150 mm, Runcorn, Cheshire, UK) after derivatization [14]. The content of cyanide was detected by the high-resolution gas chromatograph (Exactive GC, Thermo, Seattle, WA, USA) according to the national standard (GB 5009.36-2016). The concentration of EC was detected by gas chromatography–mass spectrometry (GC-MS) (Thermo, Seattle, WA, USA) after solid phase extraction [14].

### 2.7. The Expression of ureABC and CNH

*UreABC* and *CNH* genes were expressed by fusion with an MBP label using pMAL as a vector in *E. coli* BL21(DE3) strains, respectively [37,38,39]. Additionally, pMAL-*ureABC* was expressed with 1 mM IPTG in LB medium added 10.0 mg/L urea for 72 h at 25 °C. After ultrasonic disruption, the supernatant was purified by Ni-chelating affinity chromatography through His-tag that existed in N-terminus of UreC. Also, pMAL-CNH was expressed with 1 mM IPTG in LB medium added 2.0 mg/L cyanide for 72 h at 20 °C [21]. After ultrasonic disruption, the supernatant was purified by Ni-chelating affinity chromatography through His-tag that existed in N-terminus of CNH.

### 2.8. The Simulation of Chinese Liquor Fermentation

A total of 35 g of intact sorghum, 15 g of smashed sorghum and 40 mL of boiled water were mixed and sealed at room temperature for 2 h. Then, 4.5 g of rice husk, 90 g of mixed sorghum and 4 g of rice husk were successively added from bottom to top into a 250 mL flask and steamed at 100 °C for 1 h. Also, 5 mL water was added, soaked for 10 min, and cooled down to room temperature, and then 42 g of Daqu, 0.3 g of dry yeast powder, 30 mL of bacterial suspension (final concentration: 10^6^ CFU/g) or sterilized PBS buffer were added and thoroughly stirred. The mixed materials were fermented in open for 5 days as stacking fermentation (25 °C for the first day, 30 °C for the second day, 35 °C for the third day, 40 °C for the fourth day, 45 °C for the fifth day). Finally, the samples were sealed in sterilized flasks and fermented anaerobically at 38 °C for 30 days.

### 2.9. The Simulation of Distillation for Chinese Liquor

The fermented grains after fermentation were distilled in the previous reported distilling apparatus. The condensate was 8 °C. The base liquor was collected for 10 min after boiling [14].

### 2.10. The Analysis of Flavors in Simulated Base Liquor

The simulated base liquor was diluted to an alcohol degree below 10 Vol. An 8 mL diluted sample and 3 g NaCl was added into a 15 mL sample bottle, and 10 μL of 10 mg/L 2-octanol was added as internal standard. Solid phase microextraction (SPME) was carried out at 50 °C for 30 min before GC-MS analysis (Thermo, Shanghai, China) [40]. According to the ratio of the area of internal standard to the area of flavor substances, the relative concentration of flavor substances was calculated.

## 3. Results and Discussion

### 3.1. Screening of Microorganisms with the Degradative Activities of Urea

**Figure 2:**
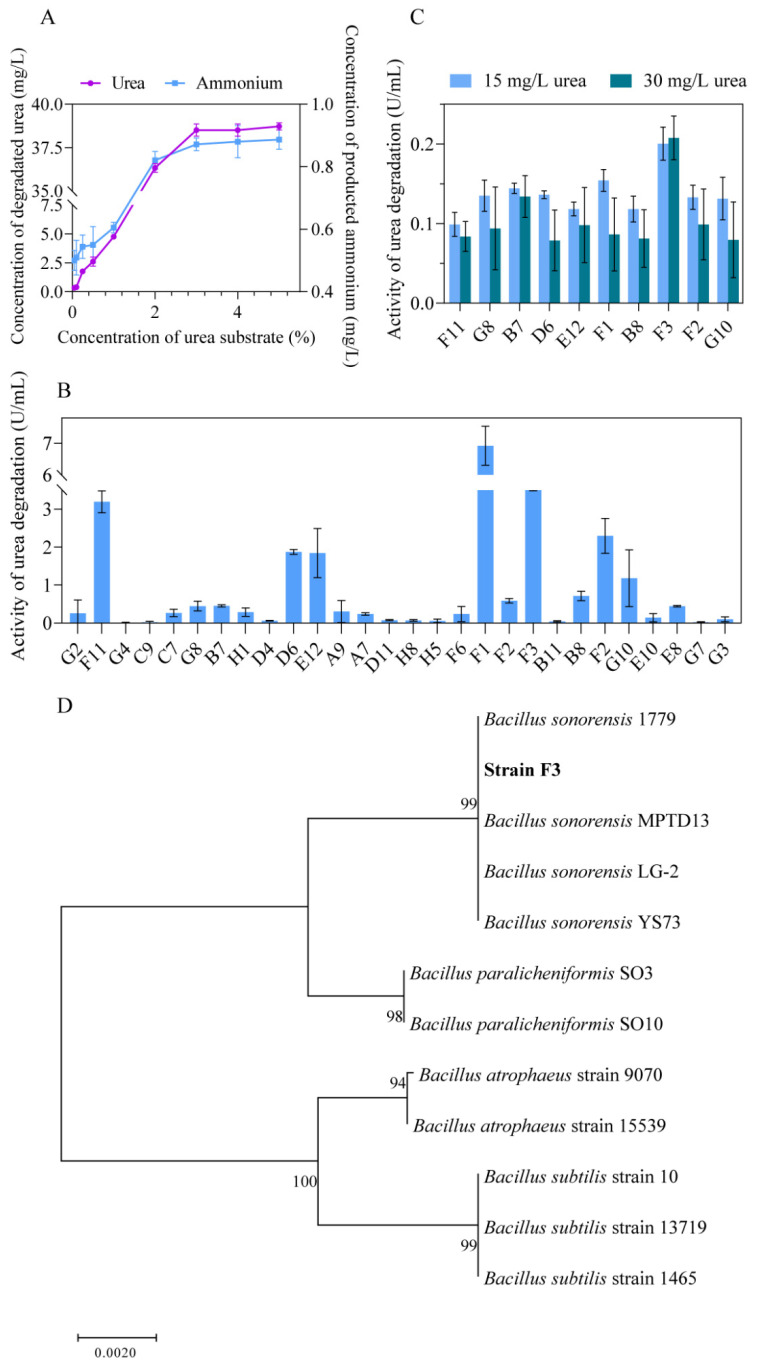
Screening of microorganisms with the degradative activities of urea. (**A**) Degradation of urea and production of ammonium at different concentrations of urea. (**B**) The degradative effect of urea by 28 isolates from the primary screening. (**C**) The degradative effect of urea by 10 isolates from the secondary screening at lower concentration of urea. (**D**) The phylogenetic tree based on 16S rRNA gene sequences from F3 strain and its relatives. The evolutionary distances are in the units of the number of base differences per site. The percentage of replicate trees in which the associated taxa clustered together in the bootstrap test (1000 replicates) are shown next to the branches.

As the major precursor of EC, urea existed in the whole process of fermentation, and some microorganisms in the fermented grains were found to metabolize urea as a nitrogen source [13]. Using M9 medium with urea as the solo nitrogen source, 28 isolates with degradative capability of urea were selected from fermented grains in the primary screening. To further narrow down the range of target strains, the degradative capability of urea at saturated concentration was tested at first. The saturation concentration of urea was determined to be 4% (Figure 2A). It was found that 10 isolates had better degradative capability in 4% urea, and the highest degradative activity of urea reached 6.92 U/mL (Figure 2B). As the real urea content in fermented grains of Chinese liquor fluctuated from 0 to 30 mg/L [14], which was over a thousand times less than the saturated substrate concentration (4% urea), it was therefore necessary to examine the degradative activity at the real content of urea for the screened isolates. The results showed that one strain with the highest enzymic activity (F3 strain) was selected at the concentrations of 15 mg/L and 30 mg/L urea (Figure 2C). Furthermore, 16S rRNA sequences of the F3 strain and its relatives were obtained from GenBank, and the phylogenetic analysis revealed that the F3 strain belongs to the *Bacillus subtilis* group and has phylogenetic closeness to *Bacillus sonorensis* (Figure 2D). The F3 strain was identified as *Bacillus sonorensis* (Genbank accession no. NZ_AOFM01000017), a facultative anaerobic bacterium. It is reported that *B. sonorensis* was isolated from soya sauce [41], with no pathogenic mechanism found so far [42]. Therefore, the application of *B. sonorensis* F3 to degrade urea has good food security, as it is an in situ microorganism isolated from fermented grains.

### 3.2. The Influence Factors of Urea Degradation for F3 Strain

**Figure 3:**
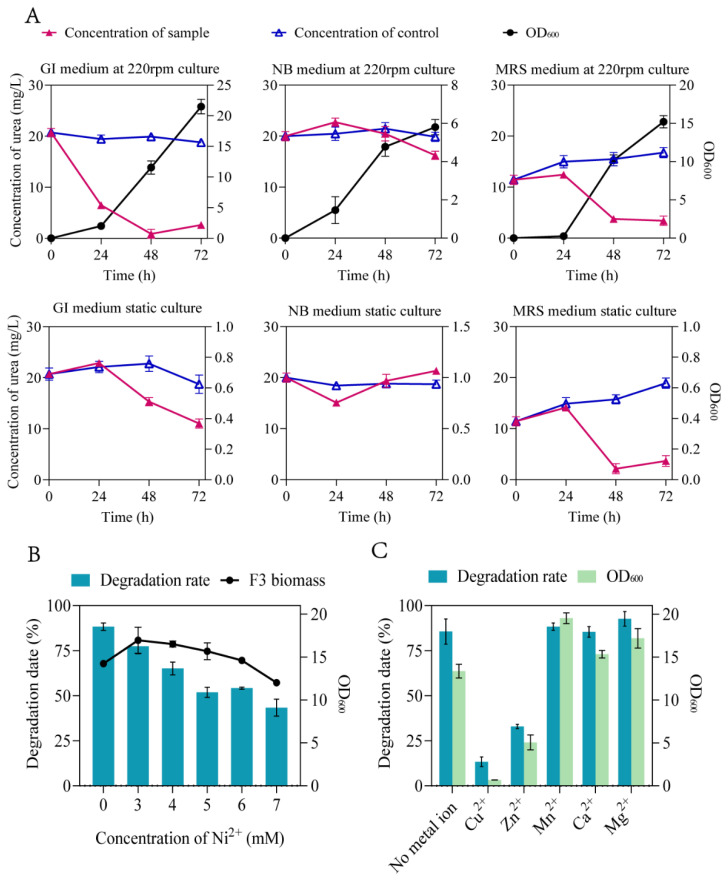
Degradation of urea by F3 strain in different conditions. (**A**) Degradation of urea in different cultural conditions. (**B**) Degradation of urea with the addition of different concentration of Ni^2+^. (**C**) Degradation of urea with the addition of different metal ions.

To further verify the degradative effect of *B. sonorensis* F3 strain on urea and optimize the conditions for the following simulated solid-state fermentation, the shaking-flask culture and static culture were performed using NB, MRS and GI medium at 10 mg/L urea concentration, respectively. The results showed that the highest degradative rate of urea could reach 95.72% at 48 h when *B. sonorensis* F3 was cultured at 37 °C with 220 rpm in the GI medium (Figure 3A).

It was reported that the existence of nickel was essential for the activities of urease in several urea-degrading microorganisms [43,44]. Thus, to verify whether the degradation of urea by *B. sonorensis* strain is dependent on the nickel or other metal ions, the degradative effect of F3 strain was tested in GI medium with Ni^2+^ and different metal ions. The results showed that the degradative rate of urea was higher for the group without Ni^2+^ (Figure 3B), indicating that the urease expressed by *B. sonorensis* F3 strain is a nickel independent enzyme and can be used to degrade urea with a higher guarantee for food safety. Furthermore, beyond Ni^2+^, the degradative rates of urea were significantly decreased when most of metal ions were added (Mg^2+^, Ca^2+^, Mn^2+^ and Zn^2+^) (Figure 3C). Therefore, the optimal cultural condition for *B. sonorensis* F3 is the utilization of the GI medium without adding metal ions.

### 3.3. Identification of Urea-Degradation Enzyme in F3 Strain

**Figure 4:**
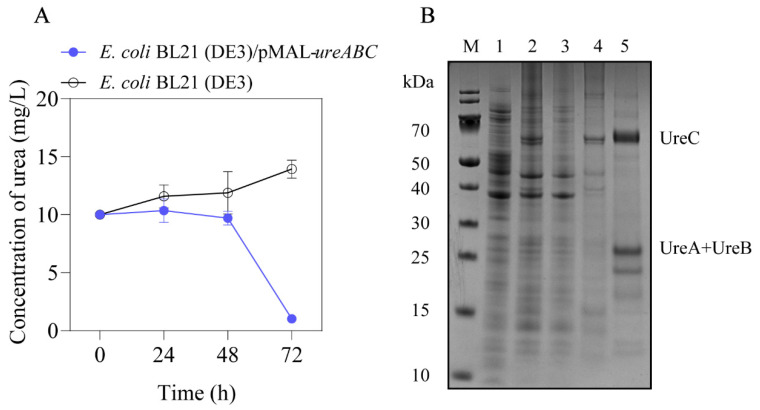
Verification of urea-degradation enzyme in the F3 strain. (**A**) Degradation of urea by the *E. coli* BL21(DE3)/pMAL-*ureABC* strain and the control (*E. coli* BL21(DE3) strain), respectively. (**B**) The analysis of SDS-PAGE. Lane M is the protein markers (Lane 1: whole BL21(DE3) sample; Lane 2: whole *E. coli* BL21(DE3)/pMAL-*ureABC* sample; Lane 3: the supernatant of *E. coli* BL21(DE3)/pMAL-*ureABC* lysate; Lane 4: the sediment of *E. coli* BL21(DE3)/pMAL-*ureABC* lysate; Lane 5: the purified UreABC enzyme).

According to the classification result of the F3 strain and the homologous sequences of urease in *B. sonorensis* from the NCBI database (Genbank accession no. NZ_AOFM01000009), the primers were designed to amplify the urease gene from the genome of the F3 strain. The sequencing results showed that this gene is a full-length 2395 bp cluster encoding UreC, a large subunit containing 529 amino acids, UreB, a small subunit containing 106 amino acids, and UreA, another subunit containing 125 amino acids, respectively. To examine the activity of UreABC from the F3 strain, the *E. coli* BL21(DE3) host harboring the pMAL-*ureABC* plasmid was constructed. The degradative rate of urea by the *E. coli* BL21(DE3)/pMAL-*ureABC* strain reached 92.64% after 72 h, while the BL21(DE3) control strain cannot degrade urea (Figure 4A). The analysis of SDS-PAGE showed that the specific bands at the size of 58 kDa and 26 kDa are consistent with the theoretical molecular weights of UreC subunit (57.3 kDa) and the UreB and UreA complexes (25.3 kDa), respectively (Figure 4B). Since the His_6_ tag only exists in the N-terminal of UreC, the specific bands of three subunits were observed after purification. The result indicates that the UreA, UreB and UreC subunits could self-assemble to form an UreABC complex, and this complex was successfully expressed in *E. coli* BL21(DE3). In addition, the degradative rate of urea was only slightly different (3.08%) between the *E. coli* BL21(DE3)/pMAL-*ureABC* strain (92.64%) and the F3 strain (95.72%), and it was proven that the urease encoded by the *ureABC* gene cluster is the key functional enzyme in *B. sonorensis* F3 to degrade urea.

### 3.4. Screening of Microorganisms with the Degradative Activities of Cyanide

**Figure 5:**
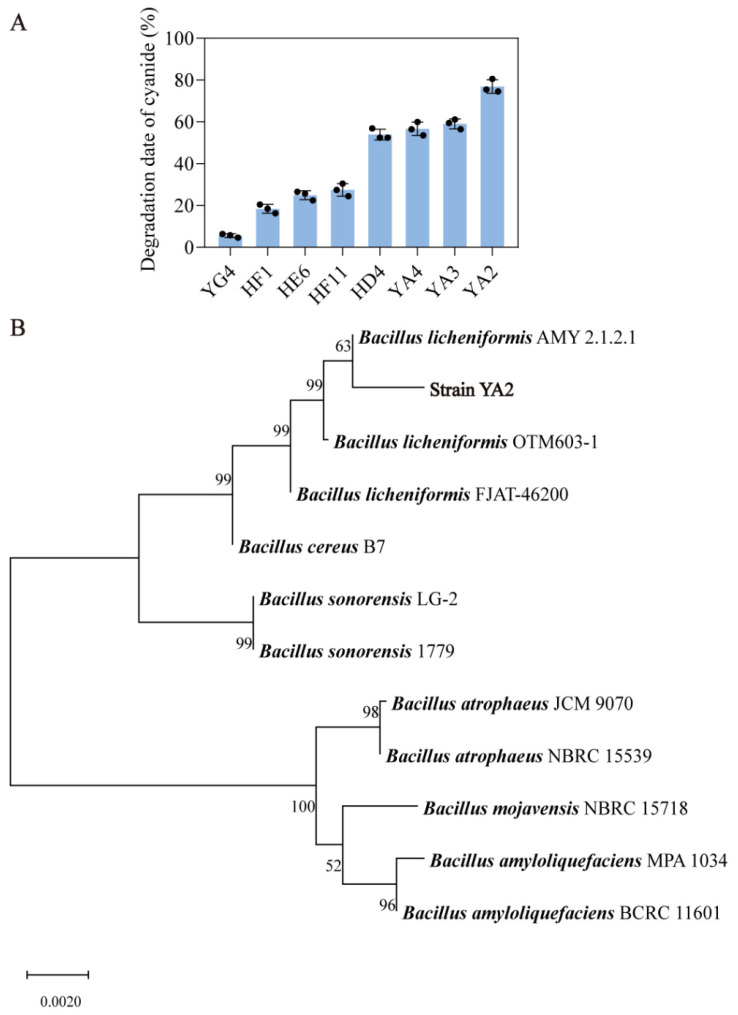
(**A**) The degradative rate of cyanide by 8 isolates with the degradative activities. (**B**) The phylogenetic tree based on 16S rRNA gene sequences from YA2 strain and its relatives.

Cyanide is another main precursor of EC in alcoholic beverages [6]. To expand the possibility of obtaining the target cyanide-degrading strains from fermented grains, both aerobic and anaerobic cultural methods were used to enrich the target strains using the minimal medium with decreasing cyanide as a solo nitrogen source. A total of four aerobic microorganisms and four anaerobic microorganisms that could degrade cyanide were isolated. After the fermentation of eight isolates in the medium containing 2.0 mg/L cyanide, the highest degradative rate (75.48%) was obtained by the anaerobic YA2 strain (Figure 5A). Additionally, 16S rRNA sequences of the YA2 strain and its relatives were obtained from GenBank, and phylogenetic analysis revealed that the YA2 strain belongs to the *Bacillus subtilis* group and has phylogenetic closeness to *Bacillus licheniformis* (Figure 5B). The YA2 strain was identified to be *Bacillus licheniformis* (Genbank accession no. KT719913) and would be further used to verify its degradative effect.

### 3.5. Identification of Cyanide-Degradation Enzyme in YA2 Strain

**Figure 6:**
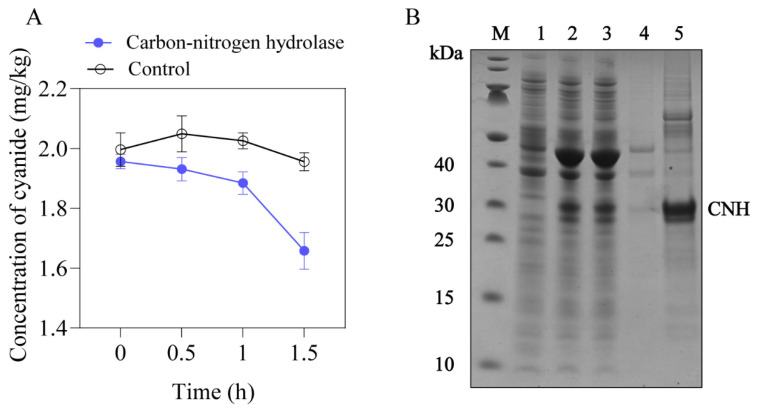
Verification of cyanide-degradation enzyme in YA2 strain. (**A**) The degradation of cyanide by purified CNH. (**B**) The analysis of SDS-PAGE (Lane M: standard protein markers; Lane 1: whole *E. coli* BL21(DE3) sample; Lane 2: whole *E. coli* BL21(DE3)/pMAL-CNH sample; Lane 3: the supernatant of *E. coli* BL21(DE3)/pMAL-CNH lysate; Lane 4: the sediment of *E. coli* BL21(DE3)/pMAL-CNH lysate; Lane 5: the purified CNH).

The information regarding homologous enzymes that might be involved in the degradation of cyanide from *B. licheniformis* was obtained from the NCBI database. A carbon-nitrogen hydrolase (CNH) was amplified from the genome of *B. licheniformis* YA2 (Genbank accession no. NZ_CP014842). It is an open reading frame encoding a protein containing 260 amino acids. The *E. coli* BL21(DE3) host harboring pMAL-CNH plasmid was constructed. The analysis of SDS-PAGE showed that there is a specific band at the size of 30 kDa, which is consistent with the theoretical molecular weights of CNH (29.1 kDa) (Figure 6B). The purified soluble protein presented the degradative ability of cyanide during the reaction with 2.0 mg/L cyanide, and the degradative rate of cyanide could reach 15.22% at 1.5 h (Figure 6A), which proved that CNH is a functional enzyme for *B. licheniformis* YA2 to degrade cyanide. The degradative rate of cyanide by *E. coli* BL21(DE3)/pMAL-CNH strain was five-fold lower than the rate by YA2 strain (75.48%), indicating that the degradative effect of CNH may have a synergistic effect with other complex enzymes or need other cofactors.

### 3.6. Elimination of EC in the Simulated Fermentation and Distillation of Chinese Liquor by F3 or YA2 Strain

**Figure 7:**
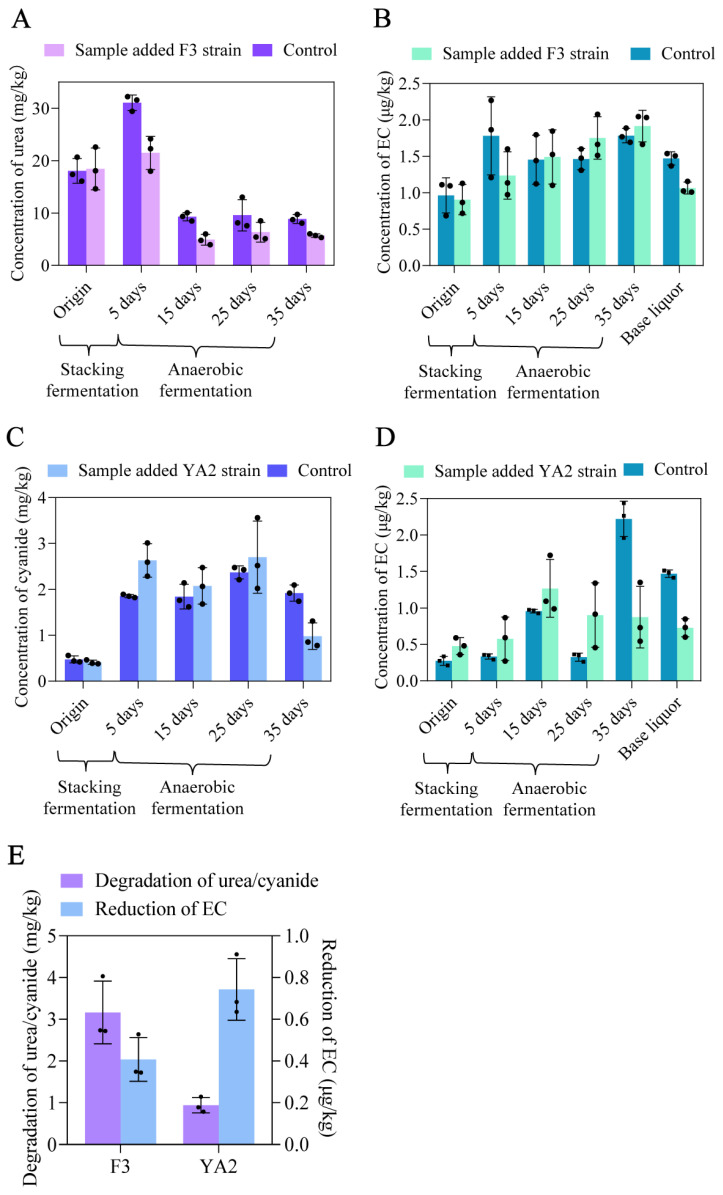
The concentration of urea (**A**) and EC (**B**) during the simulated fermentation and distillation of Chinese liquor with the addition of F3 strain. The concentration of cyanide (**C**) and EC (**D**) during the simulated fermentation and distillation of Chinese liquor with the addition of YA2 strain. (**E**) Quantitative relationship between the reduction in EC and the degradation of its precursors.

The metabolism of microorganisms greatly varies between the liquid-state and solid-state fermentations. Therefore, it is necessary to verify whether F3 and YA2 strains have degradative effects on the precursors to EC during the solid-state fermentation. In this study, a simulated solid-state system combined with the distillation system was established. The results showed that the addition of F3 strain exhibited the degradative activity of urea in the whole process of solid-state fermentation. The highest degradative rate of urea presented in the early stage of anaerobic fermentation, reaching 53.03%, and the degradative rate of urea at the end of fermentation was 35.73% (Figure 7A). Moreover, synergistic fermentation with *B. sonorensis* F3 can reduce the concentration of EC by 27.70% in the base liquor, decreasing from 1.47 to 1.06 μg/L (Figure 7B). These results indicated that *B. sonorensis* F3 successfully reduced EC in the base liquor by degrading the urea in fermented grains.

In solid-state fermentation, *B. licheniformis* YA2 did not show the degradative activity of cyanide in the early stage of fermentation, whereas the cyanide in the fermented grains of the YA2 group was reduced by 48.90%, decreasing from 1.92 to 0.98 mg/kg at the end of anaerobic fermentation (Figure 7C). This result may be caused by the requirement of a longer adaptative period for the anaerobic *B. licheniformis* YA2 to degrade cyanide. In addition, the addition of the YA2 strain can reduce the content of EC by 50.51% in the base liquor, decreasing from 1.47 to 0.73 μg/L (Figure 7D). These results indicated that *B. licheniformis* YA2 reduced EC in the base liquor by degrading cyanide.

On the whole, the addition of F3 strain reduced 0.41 μg/L EC by degrading 3.16 mg/kg urea, while the addition of YA2 strain reduced 0.74 μg/L EC by degrading 0.94 mg/kg cyanide (Figure 7E). Compared with urea, the amount of degraded cyanide was lower, but a higher degree of EC reduction was achieved. It indicated that cyanide is the main precursor of EC formation during Chinese liquor distillation; hence, the control of cyanide content in the fermented grains is a key issue in eliminating EC in the base liquor.

In previous research, several microorganisms that can reduce and control the formation of EC or its precursors have been isolated and successfully applied. Synergistic fermentation with *Bacillus amyloliquefaciens* JP21 isolated from fermented grains can reduce 50.05% of urea and 30.16% of EC in the fermented grains, respectively [45]. Synergistic fermentation with *B. licheniformis* DX530 isolated from fermented grains decreases 10% of urea and 16% of EC in the fermentation system [46]. *L. sphaericus* MT33 reduced 28.15% of urea and 41.77% of EC in the fermentation system, respectively [13]. The previous research has reduced and controlled EC in the fermentation grains, whereas reducing and controlling EC in the base liquor is the ultimate goal. In this study, by adopting the distillation process after the simulated fermentation of Chinese liquor and detecting the content of EC in the base liquor, it was proven that both *B. sonorensis* F3 and *B. licheniformis* YA2 could reduce and control the formation of EC in the base liquor. In addition, synergistic fermentation with *B. licheniformis* YA2 had a relatively better reduction effect on EC (50.51%) than the above.

### 3.7. The Degradative Activities by Mixed Culture of F3 and YA2 Strains

**Figure 8:**
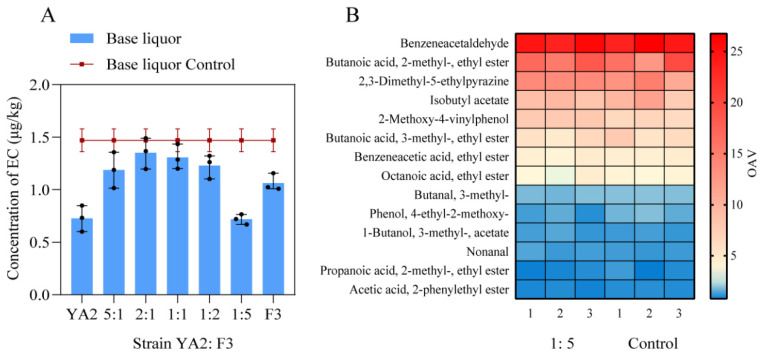
The results of fermentation of mixed culture; (**A**) The elimination of EC by the addition of YA2 and F3 strains at a different ratio. (**B**) The comparison of 14 main flavor substances in the base liquor after adding YA2 and F3 strains at the ratio of 1:5 and the control group (adding equal sterilized PBS buffer).

To explore whether the degradative metabolism of YA2 and F3 strains interact each other, the mixed cultures of two strains of different proportions (YA2:F3 = 5:1, 2:1, 1:1, 1:2, 1:5) were added to the simulated fermentation of the Chinese liquor, respectively. The results showed that YA2 strain–F3 strain = 1:5 group reduced 51.10% of EC more than the control group (adding equal sterilized PBS buffer) in the base liquor, which is the best effect among the mixed culture samples (Figure 8A).

### 3.8. The Comparative Analysis of Flavor Substances in the Base Liquor after Adding YA2 and F3 Strains

To investigate the effect of functional strains on the flavor of liquor, a comparative analysis of flavor substances in the simulated base liquor after adding YA2 and F3 strains at a ratio of 1:5 and the control group (adding equal sterilized PBS buffer) was performed. The results indicated 75 kinds of flavor substances were detected from the base liquor of the both groups, including pyrazines, alcohols, phenols, esters, organic acids, aldehydes and ketones. The odor activity value (OAV) was defined as the ratio of flavor substance concentration to its aroma threshold. The larger the OAV is, the higher the aroma contribution to the flavor of the base liquor [40]. Additionally, 14 kinds of flavor substances with higher OAV (>1.0) were comparatively analyzed as the key components of flavor in the base liquor (Figure 8B). Compared with the base liquor fermented with target strains (YA2:F3 = 1:5) with the control group, there was no significant difference in the OAV of the 14 flavor substances (*p* > 0.05), indicating that the addition of YA2 and F3 strains did not affect the main flavor substances in the base liquor. Therefore, the application of YA2 and F3 strains is feasible in terms of eliminating EC in Chinese liquor without affecting the main flavor.

## 4. Conclusions

In conclusion, to eliminate the content of EC in liquor by degrading its precursors, a *B. sonorensis* F3 strain capable of degrading urea and a *B. licheniformis* YA2 strain capable of degrading cyanide were isolated from the fermented grains of the Chinese liquor through multiple rounds of high-throughput screening. The degradative abilities of urea and cyanide reached 95.72% and 75.48%, respectively. In addition, the urease encoded by *ureABC* from the *B. sonorensis* F3 strain and the carbon-nitrogen hydrolase from *B. licheniformis* YA2 strain were identified as the key functional enzymes for the degradation of urea and cyanide, respectively. The urease from the *B. sonorensis* F3 strain is a nickel independent enzyme which could be applied for a higher guarantee of food safety.

Through synergistic fermenting with the *B. sonorensis* F3 strain, 35.73% of urea was degraded at the end of fermentation, and 27.70% of EC was eliminated in the base liquor. Through synergistic fermenting with the *B. licheniformis* YA2 strain, 48.90% of cyanide was degraded at the end of fermentation and 50.51% of EC was eliminated in the base liquor. Then, synergistic fermenting with the mixed culture of *B. sonorensis* F3 and *B. licheniformis* YA2 strains at a ratio of 5:1 saw 51.10% of EC being eliminated in the base liquor without affecting the main flavor. These two strains obtained from this study provide a method with great applicable values for eliminating EC in Chinese liquor.

## Data Availability

The original contributions presented in the study are included in the article, further inquiries can be directed to the corresponding author.
